# Renal angina index in critically ill children as an applicable and reliable tool in the prediction of severe acute kidney injury: Two tertiary centers’ prospective observational study from the Middle East

**DOI:** 10.1097/MD.0000000000036713

**Published:** 2023-12-22

**Authors:** Ahmed S.A. Soliman, Hamdan S. Al-Ghamdi, Mohamed W. Abukhatwah, Nagla M. Kamal, Shaheen A. Dabour, Soha A. Elgendy, Jaber Alfaifi, Omar M.W. Abukhatwah, Salma A.S. Abosabie, Sara A. Abosabie, Mohammed A.M. Oshi, Jwaher Althobaity, Hanan Sakr Sherbiny, Futun A. Al-Juaid, Eman G. Abdel Rahman

**Affiliations:** a Pediatric Department, Faculty of Medicine, Benha University, Egypt; b Pediatric Department, Al-Hada Armed Forces Hospital, Taif, Kingdom of Saudi Arabia; c Pediatric Department, Faculty of Medicine, Cairo University, Egypt; d Department of Child Health, College of Medicine, University of Bisha, Bisha, Kingdom of Saudi Arabia; e Internal Medicine Department, College of Medicine, Alexandria University, Egypt; f Medical Student, Faculty of Medicine, Charité – Universitätsmedizin Berlin, Berlin, Germany; g Medical Student, Faculty of Medicine, Julius-Maximilians-Universität Würzburg, Bavaria, Germany; h Neurology Division, Gaafar Ibnauf Children’s Emergency Hospital, Khartoum, Sudan; i Pediatric Department, Zagazig University, Zagazig, Egypt; j Pediatric Department, Taif Children Hospital, Taif, Kingdom of Saudi Arabia.

**Keywords:** acute kidney injury, critically ill, children, predictor, renal angina index

## Abstract

Acute kidney damage (AKI) is a common cause of pediatric intensive care unit (PICU) admissions. Implementing a reno-protective strategy for AKI prediction can significantly enhance outcomes. The renal angina index (RAI) is a risk stratification tool used to predict severe AKI. We aim to assess the reliability and accuracy of the RAI scoring system in predicting AKI as compared to other conventional AKI markers. A prospective, observational study was conducted in the PICU of 2 tertiary medical centers in the Middle East. A total of 446 patients, aged 1-month to 14-years, without chronic kidney disease were enrolled. The RAI was calculated using the renal risk and renal injury score within the first 8 to 12 hours of admission. The accuracy of RAI was compared to changes in serum creatinine from baseline. The outcome was assessed on Day 3 for presence of AKI according to the kidney disease improving global outcome (KDIGO) criteria and associated sequelae. A positive RAI (RA+) was defined as RAI readings ≥ 8. Among the patients, 89 (19.9%) had a positive RAI within the first 8 to 12 hours of admission. The RA + group had a significantly higher occurrence of Day 3 severe AKI (KDIGO stages 2&3) compared to the RA− group (60.6% vs 4.2%, *P* < .001). The RA + group also had a significantly higher utilization of renal replacement therapy (RRT) (21.3% vs 1.1%, *P* < .001), longer mean PICU length of stay in days (11.1 ± 3.5 vs 5.5 ± 2.1, *P* < .001), and increased mortality (31.4% vs 2.8%, *P* < .001) compared to the RA− group. The RAI score demonstrated superior predictive ability for Day 3 AKI, with a sensitivity of 72%, specificity of 95%, and area under the curve (AUC) of 0.837, compared to changes in serum creatinine from baseline (sensitivity: 65%, specificity: 89%, AUC: 0.773), fluid overload (sensitivity: 43.7%, specificity: 79%, AUC: 0.613), and illness severity scores (sensitivity: 52.4%, specificity: 80.5%, AUC: 0.657). RAI proved to be a reliable and rapid bedside test for identifying critically ill children at risk of developing severe AKI. This enables physicians to implement reno-protective measures and intervene early, thereby improving prognosis.

## 1. Introduction

Acute kidney injury (AKI) is a common occurrence in pediatric intensive care unit (PICU) admissions, with a reported prevalence of approximately 30%.^[1]^ It has a significant impact on clinical decision-making, as many medications require dose adjustments in the presence of renal impairment due to their renal elimination.^[2]^ AKI is associated with high mortality rates in both adult and pediatric populations, with rates of 23.9% and 13.8%, respectively.^[3]^ The severity of AKI is directly correlated with an increased risk of death, emphasizing the need for accurate prediction and early intervention.^[4,5]^

Accurate prediction of AKI is essential for initiating appropriate renal protective strategies and timely preventive interventions. Risk stratification for AKI should be performed for all critically ill patients admitted to the intensive care unit. While the kidney disease improving global outcome (KDIGO) guidelines define AKI based on serum creatinine and urine output, serum creatinine alone is not an optimal marker for severe AKI. It has limitations such as delayed peak levels and lack of specificity for intrinsic kidney injury. Factors such as autoregulation, decreased glomerular filtration, and other non-renal factors can influence serum creatinine levels.^[6,7]^

To overcome these limitations, Goldstein and Chawla introduced the concept of the renal angina index (RAI) as an empiric clinical model for predicting AKI in critically ill patients.^[8]^ The RAI utilizes patient risk variables and early signs of injury to differentiate individuals at high risk of developing severe AKI from those at low risk. The term “renal angina” was coined to describe the indicators of renal damage, similar to angina pectoris in cardiology.^[8]^ The RAI shows promise as a potential method for early detection of AKI.^[9]^

The objective of this study is to evaluate the reliability and accuracy of the RAI scoring system in predicting AKI in a PICU setting. We aim to assess the association of RAI scores with the occurrence of severe AKI, need for renal replacement therapy, length of PICU stay, and mortality. By identifying patients at high risk of severe AKI, clinicians can implement renal protective measures and early interventions to improve patient outcomes.

## 2. Aim of the study

The aim of this study is to evaluate the validity and accuracy of the RAI as a simple and applicable tool for the early detection of AKI among PICU patients.

## 3. Patients & methods

### 3.1. Study design, ethics, and sampling

This study employed a prospective observational design to investigate children admitted to the pediatric intensive care units (PICUs) at Benha University Hospitals in Benha, Egypt, and Alhada Armed Forces Hospital in Taif, Kingdom of Saudi Arabia (KSA) over a period of 12 months. Ethical approval for the study was obtained from the ethical committee of the Faculty of Medicine, Benha University, and Al-Hada Armed Forces Hospital. Written informed consent was obtained from the parents of all enrolled patients.

### 3.2. Inclusion criteria

The study included children aged 1 month to 14 years who were admitted to the PICUs and had a minimum duration of stay of 72 hours.

### 3.3. Exclusion criteria

Patients with preexisting chronic kidney disease (CKD) and those with a hospital stay of <72 hours were excluded from the study.

### 3.4. Study sample

During the recruitment period, a total of 521 hospitalizations were identified. Among these, 75 patients were excluded based on the following criteria: 29 patients with preexisting CKD, 30 patients with a hospital stay of <72 hours, 16 cases where parental consent was not obtained. As a result, a final sample of 446 eligible patients was included in the study.

### 3.5. Methods

#### 3.5.1. Data collection.

Upon admission to the PICU, various data were collected, including anthropometric measurements, demographic parameters, admission diagnosis, co-morbidities, vital signs, urine output, other clinical data, and information on ventilatory and/or inotropic support during the PICU stay. The Pediatric Risk of Mortality (PRISM) score was also recorded as an indicator of illness severity. Basic investigations, such as complete hemogram, urea, creatinine, total protein, albumin, sodium, and potassium, were performed on Day 0. Serum creatinine levels were measured daily until Day 3 and on Day 28 after PICU admission. In cases where clinical conditions warranted, serum creatinine measurements were obtained between Day 3 and 28. The outcome on Day 28 was documented as the patient status, whether still in the PICU, discharged, or deceased.

#### 3.5.2. Serum creatinine baseline.

The baseline serum creatinine was defined as the lowest SCr value in the 3 months prior to admission.^[10]^ If this measurement was not available, estimated glomerular filtration rate (eGFR) was based on estimated creatinine clearance (eCrCl) calculated by the Schwartz equation.^[11]^

#### 3.5.3. AKI definition and classification.

The KDIGO staging criteria were used to define and classify AKI in this study.^[12]^ The stages of AKI were determined as follows:

- Stage 1: An increase in serum creatinine by 0.3 mg/dL or more (26.5 micromoles/L or more) from its baseline within 48 hours, or urine output < 0.5 mL/kg/h for 6 to 12 hours.

- Stage 2: An increase in serum creatinine to 2.0–2.9 times of baseline within the prior 7 days, or urine output < 0.5 mL/kg/h for more than 12 hours.

- Stage 3: An increase in serum creatinine to 3.0 times baseline, initiation of dialysis (considered as an outcome in our study, not stage 3), serum creatinine > 4 mg/dL (353.6 micromole/L), decreased GFR < 35 mL/min/1.73 m^2^, urine volume < 0.3 mL/kg/h for 24 hours, or anuria for more than 12 hours.

#### 3.5.4. Renal angina index.

RAI is a risk prediction tool used to assess critically ill children admitted to the PICU. It is a composite of the patient risk score and injury score at 12 hours of PICU admission. The risk score is determined based on the presence of any AKI risk factors, such as ICU admission alone (score of 1), stem cell transplantation (score of 3), or the need for ventilator and/or inotropic support (score of 5). The injury score is calculated by comparing changes in estimated creatinine clearance or percentage fluid overload (%FO) from baseline. The injury score ranges from 1 to 8, depending on the degree of estimated creatinine clearance drop or rise in %FO. RAI scores range from 1 to 40. A threshold of 8 is used to determine renal angina fulfillment, indicating a high probability of developing severe AKI within 72 hours. A score of 8 or higher is considered positive for renal angina (RA+).

Please refer to Table 1 for a detailed overview of the renal angina index scoring system.^[13,14]^

**Table 1:** Renal angina index scoring system.

Variable		Score
Acute kidney injury risk strata		
Intensive care unit (ICU) admission (moderate risk)		1
Solid organ or stem cell transplant (severe risk)		3
Mechanical ventilation or vasoactive support, or both (very high risk)		5
Clinical injury signs		
Decrease in Estimated creatinine clearance (eCrCl%)	Fluid overload (FO%)	
No change	≤ 5	1
0–<25	5–<10	2
25–<50	10–<15	4
≥ 50	≥15	8

#### 3.5.5. Consequences.

The primary outcome of this study was the occurrence of AKI on day 3, based on the KDIGO criteria, with KDIGO stages 2 and 3 considered as severe AKI.^[12]^The diagnostic utility of the RAI in predicting this outcome was compared to elevated serum creatinine from baseline on day 0, fluid overload score, and illness severity score (each score alone). Day 3 was chosen as the primary consequence for several reasons. Previous research had demonstrated a correlation between Day 3 AKI and poor patient outcomes. Furthermore, international consensus suggests that beyond this point in the PICU course, transient AKI becomes less likely to be rapidly reversed, surpassing the time frame of functional AKI (pre-renal AKI).^[15]^

The secondary consequences assessed in this study included the use of RRT, the need for and duration of mechanical ventilation, the length of stay (LOS) in the intensive care unit, and the mortality rate. These outcomes were evaluated to further understand the impact of AKI and the RAI on critical care management and patient outcomes.

## 4. Statistical analysis

The collected data were organized and analyzed using SPSS version 20 software (SpssInc, Chicago, ILL Company). Categorical data were presented as numbers and percentages, and the Chi-Square (χ^2^) test was utilized for their analysis. The Shapiro–Wilks test was employed to assess the normality of quantitative data, considering normality at a significance level (P) >0.05. Normally distributed variables were reported as mean ± standard deviation, while median and inter-quartile ranges were provided for non-parametric variables. The Mann–Whitney *U* test was used for the comparison of 2 independent groups. Receiver Operating Characteristic (ROC) curves were constructed to evaluate the validity and predictive ability of the studied variables in predicting AKI. Additionally, binary logistic regression analysis was performed to identify significant predictors. Statistical significance was defined as a *P* value >.05 for non-significance, <0.05 for significance, and less than or equal to 0.001 for high significance.

## 5. Results

### 5.1. Descriptive overview

Over a period of 12 months, a total of 505 patients were admitted, out of which 446 patients were included in the study. Fifty-nine patients were excluded due to CKD (29 patients) or a PICU stay of <72 hours (30 patients). The mean age of the included cases was 61.7 ± 23.8 months, with males accounting for 58.6% of the sample. The mean PRISM II score was 13.82 ± 7.5. The most common admission diagnoses were surgical and trauma disorders (23.7%), cardiovascular disorders (11.7%), CNS, pulmonary, and gastrointestinal disorders (10.8% each), sepsis (10.5%), and shock (9.9%). The prevalence of day 0 elevated serum creatinine from baseline was 22.6%, day 3 AKI was 23%, renal angina positive was 19.9%, and overall mortality rate was 8.5%.

### 5.2. Day 3 AKI, demographics, admission diagnosis, RAI score & outcome (Table 2)

Out of the 446 cases, 103 patients had day 3 AKI (23.3%). The AKI group did not show significant differences in terms of age, gender, body surface area, and admission diagnosis. The AKI group had a significantly higher mean RAI score (9.3 ± 2.7 vs 6.1 ± 1.8), a significantly higher proportion of RAI score positive cases (64.1% vs 2.9%), increased use of inotropes (47.5% vs 16%), higher prevalence of mechanical ventilation (43.6% vs 22.1%), longer mean LOS in the PICU (10.7 ± 3.1 days vs 5.3 ± 1.2 days), higher utilization of dialysis (17.5% vs 1.5%), and significantly higher mortality (17.8% vs 5.8%).

**Table 2:** Demographics, admission diagnosis, renal angina index (RAI), and outcome of day 3 AKI.

Variable	AKI (103 patients)	No AKI (343 patients)	*P* value
Demographics			
Male n (%)	58 (56.3%)	181 (52.8%)	.5771
Age (mo)	88.9 + 14.6	70 + 11.2	.1443
Body surface area m^2^	0.93 + 0.24	0.92 + 0.23	.1250
Admission diagnosis n (%)			
Respiratory	12 (11.6%)	36 (10.4%)	.7401
Cardiovascular	13 (12.6%)	39 (11.3%)	.6860
Neurological	9 (8.7%)	39 (11.3%)	.4806
Sepsis	10 (9.7%)	37 (10.7%)	.7942
Shock	20 (19.4%)	24 (6.9%)	.0002
Gastrointestinal	4 (3.8%)	44 (12.8)	.0508
Surgical/Trauma	30 (29%)	76 (22.1%)	.0745
Endocrinal	5 (4.8%)	31 (9%)	.1717
Others	0 (0%)	17 (4.9%)	.0212
Renal angina index (RAI)			
RAI positive n (%)	66 (64.1%)	10 (2.9%)	<.001
RAI score	9.3 + 2.7	6.1 + 1.8	<.001
Outcome n (%)			
Inotropes use	49 (47.5%)	55 (16%)	<.001
Mechanical ventilation	45 (43.6%)	76 (22.1%)	<.001
Dialysis	18 (17.5%)	5 (1.5%)	.001
PICU LOS (days)	10.07 + 3.1	5.3 + 1.2	<.001
Mortality	18 (17.8%)	20 (5.8)	<.001

AKI = acute kidney injury, LOS = length of stay, PICU = pediatric intensive care unit.

### 5.3. Comparison of RA + vs RA− in relation to primary and secondary consequences (Table 3)

Comparing the positive renal angina index score (RA+) group with the negative (RA−) group, the RA + group showed a higher incidence of sepsis (16.8% vs 8.9%) and shock (16.8% vs 8.1%) at admission. The RA + group had a highly significant occurrence of total day 3 AKI (74.1% vs 10.6%) and severe AKI on day 3 (60.6% vs 4.2%), based on KDIGO stage 2 and 3 (*P* < .001). Additionally, the RA + group had significantly worse outcomes compared to the RA− group, including higher use of inotropes (61.7% vs 13.7%), mechanical ventilation (61.7% vs 18.4%), need for RRT (21.3% vs 1.1%), longer mean LOS in the PICU (11.1 ± 3.5 days vs 5.5 ± 2.1 days), and higher mortality (31.4% vs 2.8%).

**Table 3:** Demographics, admission diagnosis, and outcome in renal angina index (RAI) positive and negative cases.

	RAI positive 89 patients	RAI negative 357 patients	*P* value
Demographics			
Male n (%)	41 (46%)	198 (55.4%)	.7940
Age (mo)	87.8 + 15.8	65.5 + 13.6	.1789
Body surface area m^2^	0.91 + 0.23	0.93 + 0.25	.0321
Admission diagnosis n(%)			
Respiratory	4 (4.4%)	44 (12.3%)	.0894
Cardiovascular	13 (14.6%)	39 (10.9%)	.1043
Neurological	9 (10.1%)	39 (10.9%)	.7388
Sepsis	15 (16.8%)	32 (8.9%)	**.0041**
Shock	15 (16.8%)	29 (8.1%)	**.0015**
Gastrointestinal	4 (4.4%)	44 (12.3%)	.0894
Surgical/Trauma	16 (17.9%)	90 (25.7%)	.7023
Endocrinal	0 (0%)	36 (10%)	**.0046**
Others	0 (0%)	17 (4.7%)	.0567
Outcome n (%)			
Day 3AKI Stage 1 Day 3AKI Stage 2 Day 3AKI Stage 3 Severe Day 3AKI (stages 2&3) Total (all stages)	12 (13.4%) 31 (34.8%) 23 (25.8%) 54 (60.6%) 66 (74.1%)	23 (6.4%) 15 (4.2%) 0 (0%) 15 (4.2%) 38 (10.6%)	**.0047** **<.001** **<.001** **<.001** **<.001**
Inotropes use	55 (61.7%)	49 (13.7%)	**<.001**
Mechanical Ventilation	55 (61.7%)	66 (18.4%)	**<.001**
Dialysis	19 (21.3%)	4 (1.1%)	**<.001**
PICU LOS (days)	11.1 + 3.5	5.5 + 2.1	**<.001**
Mortality	28 (31.4%)	10 (2.8%)	**<.001**

AKI = acute kidney injury, LOS = length of stay, PICU = pediatric intensive care unit, RAI = renal angina index.

### 5.4. RAI score performance in comparison with GFR, fluid overload, and illness severity scores (Table 4 & Fig. 1)

The performance of the RAI score was assessed in comparison to the GFR, fluid overload score, and illness severity score in predicting day 3 AKI. The results demonstrated that the RAI score outperformed the other scores in terms of sensitivity, specificity, positive predictive value (PPV), and negative predictive value (NPPV).

**Table 4:** Comparative prediction of AKI Day 3 based on serum creatinine, fluid overload, and illness severity scores.

	Sensitivity	Specificity	PPV	NPPV	AUC
RAI	72%	95%	83%	91%	0.837
Day 0 Elevated Serum Creatinine (Day 0 AKI)	65%	83.5%	65%	89.5%	0.773
FLUID Overload > 5% Alone	43.7%	79%	38.5%	75%	0.613
Illness Severity Score	52.4%	80.5%	42.7%	87.7%	0.657

AKI = acute kidney injury, AUC = area under the curve, NPPV = negative predictive value, PPV = positive predictive value, RAI = renal angina index.

**Figure 1.**
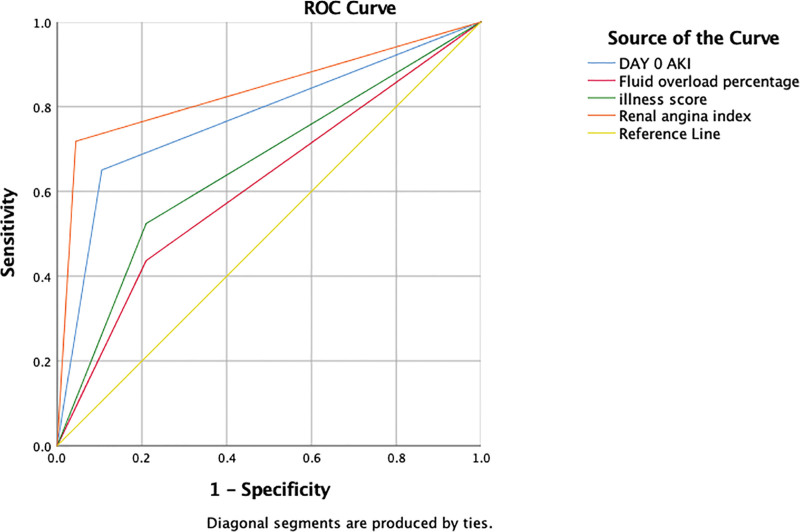
Receiver operating characteristic (ROC) Curves. The figure illustrates the ROC curves for the prediction of day 3 acute kidney injury (AKI). The different curves represent various predictive models: RAI combined with FO% (red), PRISM II score (green), RAI score alone (orange), day 0 AKI based on elevated serum creatinine from baseline (blue), and the reference line (yellow).

For the prediction of day 3 AKI, the RAI score showed a sensitivity of 72% and a specificity of 95%. This indicates that the RAI score was able to correctly identify 72% of the patients who developed AKI and accurately exclude 95% of the patients who did not develop AKI. The PPV of the RAI score was 83%, which indicates the probability of a positive RAI score correctly predicting the occurrence of AKI. The NPPV was 91%, indicating the probability of a negative RAI score correctly ruling out the presence of AKI. The area under the curve (AUC) for the RAI score was 0.837, which further confirms its superior predictive ability.

In comparison, the day 0 GFR score alone demonstrated a sensitivity of 65% and a specificity of 89%. The PPV and NPPV for the day 0 GFR score were 64% and 85% respectively. The AUC for the day 0 GFR score was 0.773.

The sensitivity and specificity of the fluid overload score (FO%) were 43.7% and 79% respectively. The PPV and NPPV for the FO% were 38% and 75% respectively. The AUC for the FO% was 0.613.

Lastly, the illness severity score (PRISM II) exhibited a sensitivity of 52.4% and a specificity of 80.5%. The PPV and NPPV for the illness severity score were 42.7% and 87.7% respectively. The AUC for the illness severity score was 0.657.

Overall, the results indicate that the RAI score demonstrated superior performance compared to the GFR, fluid overload, and illness severity scores in predicting day 3 AKI. Its higher sensitivity, specificity, and predictive values highlight its effectiveness as a predictive tool in identifying patients at risk of developing AKI.

## 6. Discussion

AKI is a prevalent condition observed in PICU admissions, with a prevalence of approximately 30%.^[15]^ In this study, we chose day 3 AKI as the primary outcome measure due to its strong association with poor secondary outcomes, such as increased PICU duration of stay, use of inotropes, mechanical ventilation, RRT, and mortality.^[14]^ Day 3 AKI also represents a point in the PICU course beyond which transient AKI is unlikely to be rapidly reversed.^[16]^

While the KDIGO guidelines define AKI based on serum creatinine levels, which is an imprecise marker for severe AKI, novel AKI biomarkers are emerging. However, their use may be limited due to cost considerations. Therefore, it is crucial to predict AKI or stratify the risk of kidney damage in order to initiate preventive measures. An appropriate risk assessment for AKI is necessary for every patient admitted to the intensive care unit.^[17]^

In the clinical context of heart disease risk factors combined with physical symptoms of coronary vasospasm (Prinzmetal Angina) in acute chest syndrome, there is a heightened likelihood of myocardial infarction before biomarkers testing. In such cases, clinicians monitor electrographic and confirmatory biomarker tests to optimize the prediction and detection of a heart attack. This approach, developed in the 1960s and 1970s, provided a framework for identifying novel therapeutic options for a disease process with a high fatality rate. With advancements in coronary bypass surgery, thrombolytic treatment, and small vessel stenting, more patients now have a higher chance of survival, and this framework continues to guide therapeutic decisions today.^[18]^

Recently, the RAI has been introduced as a potential tool for early detection of AKI and risk stratification. It is based on the available AKI epidemiology in specific pediatric populations, such as PICU admissions and organ transplant patients, along with the use of inotropes, mechanical ventilation, and signs of kidney injury, such as changes in estimated creatinine clearance from baseline and FO%. Fluid overload has been associated with increasing morbidity and mortality, with a 3% increase in mortality for every 1% increase in fluid overload.^[19]^ The RAI, incorporating these factors, has shown improved predictive ability for day 3 AKI compared to using each score alone at admission.^[14]^ Furthermore, the RAI has outperformed other traditional markers of renal injury, indicating increased predictive accuracy.^[9]^

This prospective observational study conducted in 2 tertiary centers, Benha University Hospital in Egypt and Alhada Armed Forces Hospital in the Kingdom of Saudi Arabia, included 446 children admitted to PICU units. The study aimed to assess the association between day 3 AKI and increased morbidity and mortality in critically ill children. Additionally, it sought to determine the predictive ability of the RAI risk stratification system early in the PICU course as an easily applicable bedside tool for predicting day 3 AKI and compare its performance with that of elevated serum creatinine, fluid overload, and illness severity scores, aiming to improve predictive precision and accuracy for significant severe AKI, thereby aiding clinicians in making better management decisions.

In our study, we observed an incidence of AKI on day 3 in 23.3% of cases. Among the patients, 19.9% had a positive Risk Assessment for AKI (RAI) score (>8) at admission on day 0, which was significantly associated with a higher occurrence of day 3 AKI. Furthermore, the RAI-positive group (RA+) exhibited a prolonged duration of hospital stay, higher rates of mechanical ventilation, inotropic support, RRT utilization, and mortality. These findings align with previous studies by Menon et al^[9]^ and Basu et al,^[18]^ which reported similar associations between RAI positivity at PICU admission, day 3 AKI, and adverse outcomes. Raina Kaur et al also observed a significantly higher incidence of day 3 AKI in patients with RAI > 8 (RA + group), with 75% of day 3 AKI cases belonging to the RA + group. Moreover, both day 3 AKI and RA + were associated with higher PRISM II scores (indicating greater illness severity), increased rates of inotropic support, mechanical ventilation, RRT utilization, and higher mortality rates.^[20]^ Collectively, our study and these previous investigations highlight a strong relationship between day 3 AKI and higher RAI scores.

Furthermore, our results demonstrate that the RAI score on day 0 exhibited superior predictive ability compared to other markers, such as increased serum creatinine from baseline, FO%, and illness severity score (PRISM II), for clinically significant AKI. The RAI showed improved sensitivity of 12%, 29.7%, and 19.6%, respectively, in correctly predicting AKI at any stage, and 60.6% for severe AKI, whereas the use of increased serum creatinine from baseline alone predicted only 36.6% of severe AKI cases. Additionally, the RAI showed high effectiveness in ruling out children at low risk of AKI, with a specificity of 95% and a NPPV of 91%. These findings are consistent with the results of the AWARE study,^[18]^ Basu et al,^[14]^ and other relevant studies,^[20–22]^ which also demonstrated that the RAI outperformed traditional markers of renal injury and exhibited increased predictive accuracy.

Early detection of severe AKI can potentially improve patient prognosis. Previous treatment studies have primarily focused on patients with severe and advanced AKI enrolled in intensive care unit (ICU) programs. Surprisingly, patients with severe AKI 3 days after hospital admission had worse outcomes across all evaluated metrics, even when their initial condition at admission was not significantly worse than that of patients without AKI. The RAI may enable tailored trial designs for therapy based on the degree of AKI risk,^[9]^ allowing for the evaluation of treatment regimens earlier in the course of AKI as a whole. Moreover, RAI-directed biomarker assessment has the potential to enhance targeted testing methods and strengthen positive predictions for AKI. Additionally, the RAI may find utility in future studies examining other conditions, such as AKI in conjunction with CKD.

One notable strength of this study is the utilization of a sufficiently large sample size obtained from 2 tertiary care centers in different developing countries in the Middle East. This approach ensured a heterogeneous mix of AKI risk among the patients, covering a period of nearly 1 year of PICU admissions in these centers. Furthermore, the study incorporated the combined use of baseline serum creatinine and urine output criteria for defining AKI, as advocated by Kaddourah et al in their recent AWARE study. It has been shown that relying solely on plasma creatinine levels failed to identify AKI in 67.2% of patients if urine output criteria were not considered.^[23]^

However, it is important to address certain limitations of this study. Firstly, the accuracy of baseline body weight determination was compromised for patients presenting with hypovolemia (dehydration and hemorrhage), as their admission body weight did not reflect their actual baseline weight. Moreover, a significant portion of these cases lacked records of baseline body weight, particularly among younger patients who experience rapid weight changes. This limitation can affect the calculation of the FO% for these cases. Additionally, the baseline estimated GFR was derived from the admission serum creatinine since most patients did not have their baseline values available prior to admission. Another important limitation is the lack of AKI follow-up in the study, preventing the determination of the association between RAI score and AKI recovery in later stages.

We emphasize the need for future research to incorporate a modified RAI specifically designed for pediatrics. This modified approach should encompass patients with CKD and extend beyond the confines of the PICU to include non-PICU admissions. By expanding the examined groups and considering these modifications, greater accuracy and reliability can be achieved in assessing AKI.

## 7. Conclusions

In conclusion, our study demonstrates that the RAI is a straightforward and practical bedside tool that can be easily implemented to accurately predict the likelihood of severe AKI and its associated adverse outcomes. The RAI serves as a valuable guide for clinicians, enabling them to make timely and informed decisions that promote renal protective strategies and ultimately improve the overall outcome of critically ill children. Its simplicity and applicability make it a valuable addition to the clinical arsenal in managing pediatric patients in critical care settings.

## Author contributions

**Conceptualization:** Ahmed Soliman, Mohamed Abukhatwah, Eman G. Abdel Rahman.

**Data curation:** Ahmed Soliman, Mohamed Abukhatwah, Nagla M. Kamal, Shaheen A. Dabour, Soha A. Elgendy, Salma A.S. Abosabie, Sara A. Abosabie, Mohammed Oshi, Futun A. Al-Juaid, Eman G. Abdel Rahman.

**Formal analysis:** Ahmed Soliman, Jaber Alfifi, Futun A. Al-Juaid, Hanan Sherbiny.

**Investigation:** Hamdan Al-Ghamdi, Mohamed Abukhatwah, Eman G. Abdel Rahman.

**Methodology:** Hamdan Al-Ghamdi, Soha A. Elgendy.

**Project administration:** Shaheen A. Dabour, Eman G. Abdel Rahman.

**Supervision:** Eman G. Abdel Rahman.

**Validation:** Eman G. Abdel Rahman.

**Writing – original draft:** Ahmed Soliman, Mohamed Abukhatwah, Nagla M. Kamal, Shaheen A. Dabour, Soha A. Elgendy, Jaber Alfifi, Omar Abukhatwah, Salma A.S. Abosabie, Sara A. Abosabie, Mohammed Oshi, Jwaher Althobaity, Hanan Sherbiny, Eman G. Abdel Rahman.

**Writing – review & editing:** Ahmed Soliman, Mohamed Abukhatwah, Nagla M. Kamal, Shaheen A. Dabour, Soha A. Elgendy, Jaber Alfifi, Omar Abukhatwah, Salma A.S. Abosabie, Sara A. Abosabie, Jwaher Althobaity, Hanan Sherbiny, Eman G. Abdel Rahman.
